# Rehabilitation approach and results of using the biofeedback method (GIGER MD device) in children with neurogenic bladder

**DOI:** 10.3389/fneur.2023.1198232

**Published:** 2023-07-21

**Authors:** Andrea Cvitkovic-Roic, Danijel Mikulic, Daniel Turudic, Danko Milosevic, Goran Roic, Valentina Matijevic

**Affiliations:** ^1^Helena Clinic for Pediatric Medicine, Zagreb, Croatia; ^2^Faculty of Medicine, Josip Juraj Strossmayer University of Osijek, Osijek, Croatia; ^3^Faculty of Medicine, University of Rijeka, Zagreb, Croatia; ^4^Department of Emergency, University Hospital Centre “Sisters of Charity”, Zagreb, Croatia; ^5^Department of Pediatrics, University Hospital Centre Zagreb, Zagreb, Croatia; ^6^Croatian Academy of Medical Sciences, Zagreb, Croatia; ^7^School of Medicine, University of Zagreb, Zagreb, Croatia; ^8^Children's Hospital Zagreb, Zagreb, Croatia; ^9^Department of Rheumatology, Physical Medicine and Rehabilitation, University Hospital Centre “Sisters of Charity”, Zagreb, Croatia; ^10^Libertas International University, Zagreb, Croatia; ^11^School of Medicine, Catholic University of Croatia, Zagreb, Croatia

**Keywords:** GIGER MD, rehabilitation, neurogenic bladder, children, spina bifida

## Abstract

**Background:**

GIGER MD device applies a biofeedback method through stimulated coordinated rhythmic and dynamic movements of the trunk and extremities in an anti-gravity position, thus helping to regain lost motor functions.

**Methods:**

In this article, the performance of the GIGER MD device was evaluated in 36 children with neurogenic bladder measuring gait speed, voiding bladder capacity, deviation from the age-adjusted bladder capacity (measured using the Koff scale), and urinary incontinence.

**Results:**

Children using the GIGER MD device had an increase in voiding bladder capacity (33.79%, median volume increase of 50 ml) with a subsequent median decrease in median age-adjusted bladder capacity by 45.50% (median deviation before was 36% vs. 16% after treatment). The number of urinary incontinence episodes also reduced by 55.57% (7–3 episodes *per* day), and the 20-meter motor gait speed increased by 14.26% (from 23 to 19 s).

**Conclusion:**

Children who follow the GIGER MD therapy regularly for a period of 6 months show that CNS functional damage can be significantly improved.

## Introduction

Rehabilitation of children requires a multidisciplinary approach that uses brain neuroplasticity to stimulate and develop normal motor patterns of posture and movement (1–3). GIGER MD device uses biofeedback through stimulated coordinated rhythmic and dynamic movements of limbs and trunks in an anti-gravity position (4). Rhythmically and repetitively aligned movements encourage relearning of the central nervous system (CNS) functions and stimulate receptors on the skin, muscles, and joints. Repetition of such movements forms a new set of impulses which leads to the reorganization of the nervous system. Such stimulated movements help restore the motor, vegetative, and higher mental functions lost to CNS damage. Children achieve new automaticity in movements and body posture. Children exposed to neuro-risk factors who have developed clinically significant symptoms of deviation from proper psychomotor development can be directed toward normal development through a habilitation program. There is a broad spectrum of indications for GIGER MD therapy, with mainly neurological and musculoskeletal damage and diseases mentioned. To date, no contraindications have been described (5). Reports of this method are an exception in the literature. This paper, therefore, aims for a dual purpose: (1) to provide information about the existence of this method and its possibilities and (2) to present the results we achieved by applying this method in children with neurogenic bladder.

## Materials and methods

This retrospective case series included 36 children with neurogenic bladder as a common feature. Alongside neurogenic bladder, the patients also had congenital anomalies, and spinal cord and/or CNS damage. Twenty-five out of 36 children were also diagnosed with paraplegia prior to enrollment in the study. Because of the sensitivity of the GIGER MD procedure on children with neurological damage, we did not find an indication for applying this method in healthy children. For all children included in the research, measurements of 20-meter gait speed, the number of rotations performed per week, bladder capacity, and urine incontinence episodes were taken prior to enrollment and after a treatment period of 6 months. In 36 children with a diagnosis of neurogenic bladder, the parents kept a 48-h diary, both before and after the therapy, which was handed to them. Self-urinating children urinated into volume containers to obtain voided urine volume (mL), while catheterized children’s volumes were measured *via* catheter bags. Children with nocturia were woken by their parents 1 and 4 h after falling asleep and offered to urinate. Age-adjusted bladder capacity was estimated using Koff’s equation: expected capacity (mL) = [age + 1] ×30 (6). Using the aforementioned equation, we calculated the deviation from the expected bladder capacity and expressed the deviation in percentages. The number of incontinent episodes per day was monitored over a week, and the average was taken. There is no published literature data on the use of biofeedback (GIGER MD) device in patients with neurogenic bladder.

## Statistical analysis

The following variables were measured before and after GIGER MD therapy (gait speed, voiding bladder capacity, deviation from the Koff scale, urinary incontinence). Data are expressed as mean, standard deviation, median, and interquartile range (IQR). The difference between paired variables was analyzed using a two-sample paired (Wilcoxon) signed rank test. The Spearman correlation coefficient was applied to assess the strength of a monotonic relationship between paired variables (6). A correlation matrix heatmap was used to clearly show the relationships of individual groups of children for the same parameters before and after therapy. A correlation heatmap showed the graphical representation of the correlation between different variables. All applied tests were two-tailed, and *p*-values ≤0.05 were acknowledged as statistically significant. Statistical analysis was performed with the program GraphPad Prism 8.4.3.686.

## Results

A total of 36 children were enrolled in the study spanning three to 6 months. 25/36 children (A) had a gait assessment, while 36/36 (B) underwent voiding bladder capacity measurement estimation and urinary incontinence frequency. 11/36 children had no gait assessment. There was no difference in training between A and B groups.

**Figure 1:**
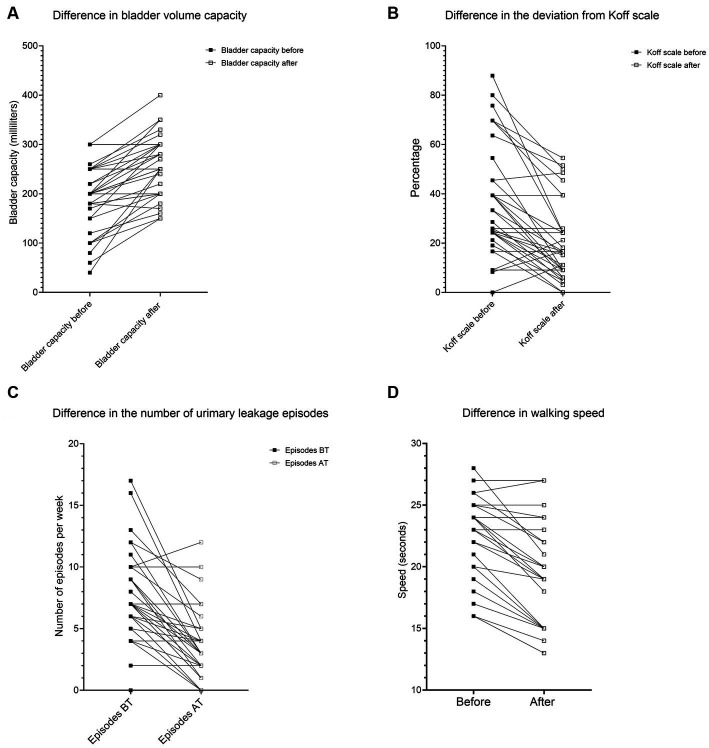
The difference in **(A)** bladder volume capacity, **(B)** deviation from the Koff scale, **(C)** gait speed, and **(D)** urinary leakage episodes.

The first group of children (A) had an age mean of 12.51 (+/− 4.1) years with a median of 12.33 years (9.792–14.50) and a male-to-female ratio of 1:2.1. The youngest child in the group was 7 years of age. The average group gait speed median before treatment measured 23 s (IQR 19.5–25), while after treatment, the median speed reduced to 19 s (IQR 15–22.5). The 20-meter gait speed after treatment increased by 14.26%, Wilcoxon signed rank test (two-tailed, *p* < 0.0001, Z-statistic 3.945). The results are shown in Figure 1.

**Figure 2:**
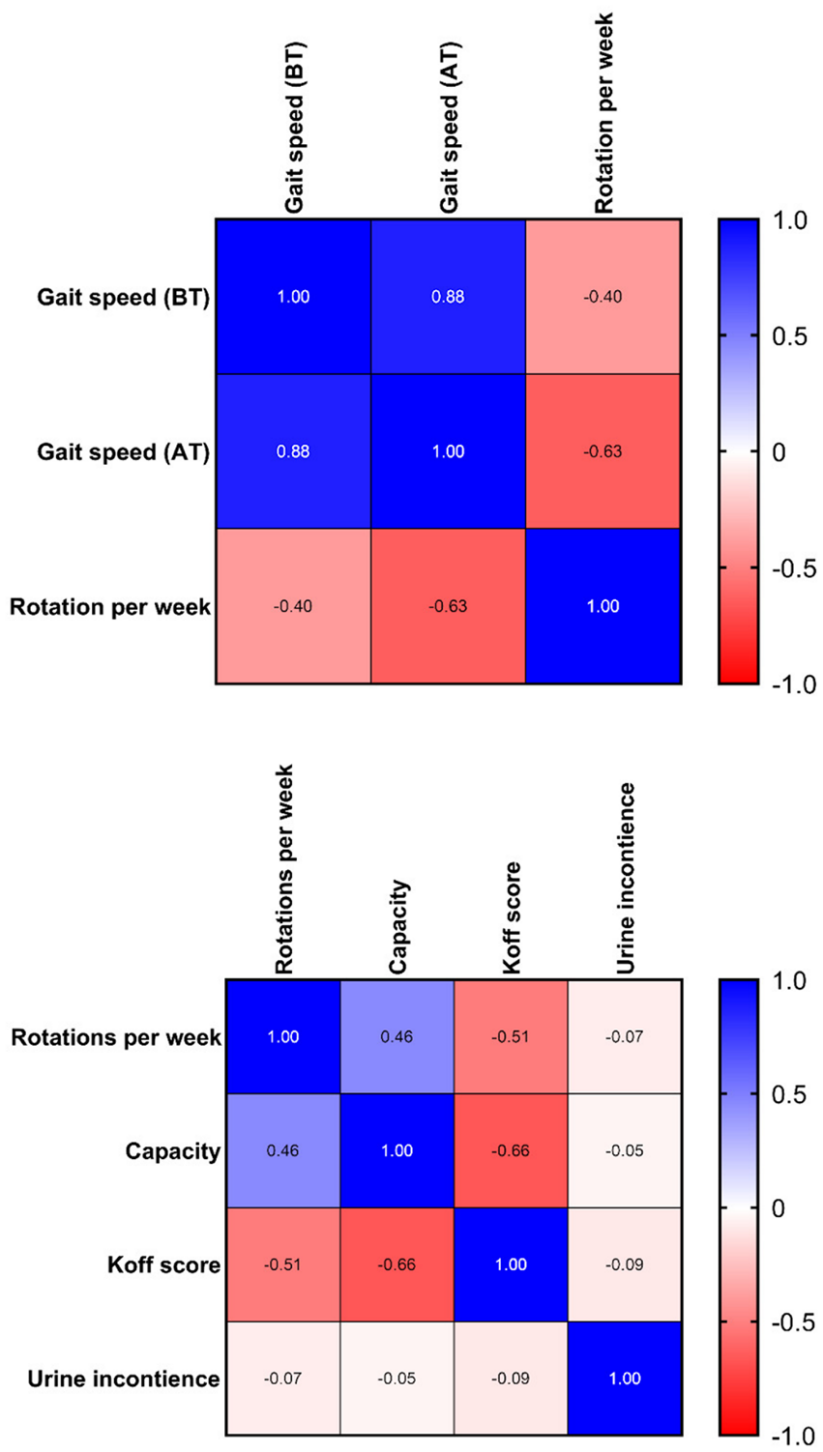
Correlation matrix of Tables 1, 2.

The second group of children (B) had an age mean of 12.15 +/− 4.51 years with a median of 11.34 years (IQR 8.790–14.19) and a male-to-female ratio of 1:2.17. The average group voiding bladder capacity median before treatment measured 200 (IQR 165–250) milliliters, while after treatment, the median volume increased to 250 (IQR 200–300) milliliters. The voiding bladder capacity after treatment increased by 33.79%, Wilcoxon signed rank test (two-tailed, *p* < 0.0001, *Z*-statistic −4.84). The results are shown in Figures 1A, 2.

**Table 1:** Age, number of rotations and the difference in gait speed, bladder capacity, Koff scale and number of urinary incontinence before and after GIGER MD therapy.

Variable	Mean (SD)	Median (IQR)
Group age (*N* = 38)	12.15 (4.5)	11.34 (8.8 to 14.19)
No. of rotations per week	1708 (545.4)	1900 (1,275 to 2,100)
Gait speed before therapy (s)	22.16 (3.6)	23 (19.50 to 25)
Gait speed after therapy (s)	19.36 (4.23)	19 (15 to 22.5)
Bladder capacity before therapy (ml)	193.2 (64.4)	200 (165 to 250)
Bladder capacity after therapy (ml)	257.4 (61.01)	250 (200 to 300)
Koff scale before therapy (percentage)	35.90 (21.87)	30.95 (23.48 to 45.45)
Koff scale after therapy (percentage)	19.58 (15.19)	16.67 (9.09 to 24.24)
No. of urinary leak episodes before therapy	6.82 (4.3)	7 (4 to 9.25)
No. of urinary leak episodes after therapy	3.11 (2.97)	3 (0.75 to 4.25)

**Table 2:** Spearman rank correlation matrix between 20-meter gait speed (seconds) and rotations per week.

*p*-values			
	Gait speed (BT)	Gait speed (BT)	Rotations per week
Gait speed (BT)		**<0.001**	0.051
Gait speed (AT)	**<0.001**		**<0.001**
Rotations per week	0.051	**<0.001**	

Correlation matrix *ρ*			
	Gait speed (BT)	Gait speed (AT)	Rotations per week
Gait speed (BT)	1	0.877	−0.395
Gait speed (AT)	0.877	1	−0.635
Rotations per week	−0.395	−0.635	1

Correlation matrix *ρ* confidence intervals			
	Gait speed (BT)	Gait speed (AT)	Rotations per week
Gait speed (BT)		0.7313 to 0.9460	−0.6900 to 0.01241
Gait speed (AT)	0.7313 to 0.9460		−0.8273 to −0.3088
Rotations per week	−0.6900 to 0.01241	−0.8273 to −0.3088	

BT, before therapy; AT, after therapy. The bold values in the table are *p*-values.

The average median deviation from the Koff scale before treatment measured 36 (IQR 24–54.5) percent, while after treatment, the median deviation decreased to 16 (IQR 9–39) percent. After treatment, the deviation from the Koff formula decreased by 45.40%, Wilcoxon signed rank test (two-tailed, *p* < 0.0001, *Z*-statistic 4.55). The results are shown in Figure 2.

The average median urinary incontinence episodes measured 7 (IQR 4–9) *per* day, while after treatment, the number of episodes decreased to 3 (IQR 1–4) per day. Episodes of urinary incontinence were reduced by 55.57%, Wilcoxon signed-rank test (two-tailed, *p* < 0.0001, *z*-statistic 4.52). The results are shown in Figure 2.

Children with gait assessment had reduced voiding bladder capacity (mean 153 +/− 65.43 ml vs. 219.1 +/− 49.72 ml, value of *p* 0.043) and had a higher deviation from the Koff scale (mean 48.31 +/− 26.24% vs. 27.81 +/− 13.88, *p* = 0.092) vs. children with no gait assessment. There were no statistical differences between other parameters (Mann–Whitney *U*-test).

Spearman’s rank correlation coefficient was used to assess the relationship between the variables. The variable of gait speed (*ρ* = −0.63) shows a strong negative correlation to the number of rotations per week. The voiding bladder capacity (*ρ* = 0.46) shows a moderate positive, while simultaneously, the decrease in deviation from the Koff scale shows a moderate negative (*ρ* = −0.51) correlation (Table 3).

**Table 3:** Spearman rank correlation matrix between voiding bladder capacity, Koff scale deviation, urinary incontinence per day (UI).

*p*-values			
	Rotations per week	Capacity	Koff score
Rotations per week		**0.004**	**0.001**
Capacity	**0.004**		**<0.001**
Koff score	**0.001**	**<0.001**	

Correlation matrix *ρ*			
	Rotations per week	Capacity	Koff score
Gait speed (BT)	1	0.458	−0.515
Gait speed (AT)	0.458	1	−0.660
Rotations per week	−0.515	−0.660	1

Correlation matrix *ρ* confidence intervals			
	Rotations per week	Capacity	Koff score
Gait speed (BT)		0.1528 to 0.6838	−0.7212 to −0.2242
Gait speed (AT)	0.1528 to 0.6838		−0.8125 to −0.4238
Rotations per week	−0.7212 to −0.2242	−0.8125 to −0.4238	

BT, before therapy; AT, after therapy. The bold values in the table are *p*-values.

## Discussion

The primary *modus operandi* of the GIGER MD® device consists of coordinated movements of arms and legs *via* hand and foot pedals, which are triggered in different phases from each other. Children who could perform gait assessment had a greater overall benefit from biofeedback therapy vs. children without gait assessment.

Spine movements are performed in the frontal, sagittal, and horizontal planes. Such stimulated movements are as follows: rotation, movement to the left and right, and elongation and shortening. In the supination position, the child alternates the following positions: basic, extension, flexion, and traction positions. A damaged neural pathway can be reorganized if rhythmic, dynamic, and symmetrical movements of the limbs and trunk activate it. The injured part of the neurological system is activated simultaneously with the activation of the surrounding healthy parts of the neurological system, whereby the damaged part learns from the healthy how to restore the lost function. The key is to activate a significantly larger area of the healthy part simultaneously with the injured part. In that way, the healthy part avoids the dominance of the damaged part. It is important to achieve reintegration and coordination of the arms, legs, hands, fingers, trunk, and head motor functions with auditory, visual, and higher mental functions (3).

Brain neuroplasticity is significantly more pronounced during growth and development in children (7). In order to restore the functions of the damaged areas of the CNS, enough appropriate impulses must arrive from the periphery to be processed and restore normal functioning. In paraplegic patients, rhythmically coordinated movements GIGER MD device stimulates the base of the brain stem, while at the same time, hand movements stimulate the cerebellum. Spiral (circular) movements of the body, in turn, activate the locomotor center in the spinal cord. Repeating such movements forms a new set of impulses that reorganizes the nervous system and achieves new automaticity in movements and body posture. This applies to patients with spinal cord injuries, cerebral palsy, Parkinson’s disease, stroke, and idiopathic scoliosis. It is also used in several other neurological conditions, such as peripheral nervous system diseases and spina bifida.

A therapeutic approach to the neurogenic bladder is a challenging one. Despite numerous (re)habilitation procedures, this sub-area has not been definitively resolved so far and belongs to the domain of future answers.

Contemporary treatment is now reduced to several recommendations (1) clean intermittent catheterization (2) reflex voiding and bladder expression with Valsalva or Credé (3) condom catheter drainage or indwelling catheters such as urethral catheters or suprapubic tube (4) urinary diversion by ileal conduit if self-catheterization is impossible (5) suppressing neurogenic detrusor overactivity or compliance alteration (anticholinergics, intra-detrusor botulinum toxin) (6) aponeurotic suburethral tape or artificial urinary sphincter for sphincter insufficiency (7) Transcutaneous Electrical Stimulation for Neurogenic Bladder Dysfunction (8) resorting to surgery is sometimes necessary either after the failure of non-invasive treatments (e.g., bladder augmentation in case of neurogenic detrusor overactivity. Resistant to pharmacological treatment) (8–12). We here present the GIGER MD method, which may be able to improve some neurogenic bladder treatment procedures. In all these circumstances GIGER MD treatment can still be performed in cases of all the above-mentioned diagnoses.

The best achievement of applying the GIGER MD technique was reducing the number of urinary incontinence episodes by 55.57%. The incontinence rate (leakage episodes/per day) shows considerable improvement in most children (Figure 1C; Table 1). This cannot be attributed to any single variable separately but probably to the combined effect of all variables, which had different recovery rates. Our sample size was inadequate for the non-parametric equivalent of the ANOVA test (Scheirer–Ray–Hare). This is a limitation of our study. We strongly encourage future research on this subject. However, 1/6 had deterioration of several urinary incontinence episodes, while 5/36 children showed no improvement despite 500–2,300 rotations.

The second-best achievement was the improvement of bladder capacity. The moderate positive correlation between bladder capacity and gait speed means that the more rotations in the Giger MD a child makes, the more the bladder capacity (in milliliters) will increase significantly. The moderate negative correlation between the Koff scale and gait speed means that the more rotations in the Giger MD a child makes, the more the Koff scale of bladder size will correspond to the values for age (measured as a percentage) (Figure 1B). The moderate correlation between gait speed and bladder capacity/Koff scale is due to the different recovery rates between these two variables. Gait speed recovery vs. bladder capacity/Koff scale shows a faster recovery rate of bladder capacity compared to gait speed. 7/36 children had no improvement despite exerting themselves on rotations (500–2,300 rotations). There was no deterioration in bladder capacity and Koff scale. Most of them had surgical anorectal interventions or congenital anomalies in that area.

The third-best is the improvement of gait speed. The strong negative correlation between rotations per week and gait speed means that the more rotations in the Giger MD a child makes, the faster the 20-meter track will be walked by a child (Figures 1D, 2). There were only 5/25 children with no improvement in gait speed. There was no deterioration in gait speed. Most had surgical treatment, congenital anomalies, and hemiparesis in that area.

## Conclusion/implications

Applying the GIGER MD method, achieving a satisfactory improvement in urinary incontinence episodes, gait speed, and bladder capacity in patients with neurogenic bladder is possible. The method is harmless, psychologically acceptable for the patient, and can be applied with other non-surgical and surgical procedures. This is the first study using a biofeedback (GIGER MD) device in patients with neurogenic bladder. Further studies are needed to evaluate the long-term effect of the biofeedback (GIGER MD) device on the neurogenic bladder. According to our experience so far, it takes 3–6 months of regular exercise and further maintenance exercise 2 times a month or more often, depending on the possibilities.

## Data availability statement

The raw data supporting the conclusions of this article will be made available by the authors, without undue reservation.

## Ethics statement

The studies involving human participants were reviewed and approved by Ethical Committee of the University Hospital Centre “Sisters of Charity.” Written informed consent to participate in this study was provided by the participants’ legal guardian/next of kin.

## Author contributions

AC-R, DMil, and VM: conceptualization. AC-R and VM: methodology. DT: software. DMil, GR, and VM: validation. DT and DMil: formal analysis, and writing—original draft preparation and review and editing. AC-R, DMik, GR, and VM: investigation. AC-R, DMik, and VM: resources. DMik and VM: data curation. DT: visualization. AC-R, GR, and VM: supervision. All authors have read and agreed to the published version of the manuscript.

## Conflict of interest

The authors declare that the research was conducted in the absence of any commercial or financial relationships that could be construed as a potential conflict of interest.

## Publisher’s note

All claims expressed in this article are solely those of the authors and do not necessarily represent those of their affiliated organizations, or those of the publisher, the editors and the reviewers. Any product that may be evaluated in this article, or claim that may be made by its manufacturer, is not guaranteed or endorsed by the publisher.
